# Simultaneous Microwave Extraction and Separation of Volatile and Non-Volatile Organic Compounds of Boldo Leaves. From Lab to Industrial Scale

**DOI:** 10.3390/ijms15057183

**Published:** 2014-04-25

**Authors:** Loïc Petigny, Sandrine Périno, Matteo Minuti, Francesco Visinoni, Joël Wajsman, Farid Chemat

**Affiliations:** 1 GREEN Extraction Team, Institut National de Recherche Agronomiques INRA, Unité Mixte de Recherche UMR 408, Avignon University, F-84000 Avignon, France; E-Mails: loic.petigny@univ-avignon.fr (L.P.); farid.chemat@univ-avignon.fr (F.C.); 2 BASF, Beauty Care Solutions France, F-69007 Lyon, France; E-Mail: joel.wajsman@basf.com; 3 Milestone srl, Via Fatebenefratelli 1/5, I-26010 Sorisole, Bergamo, Italy; E-Mails: m.minuti@milestonesrl.com (M.M.); f.visinoni@milestonesrl.com (F.V.)

**Keywords:** boldo, microwave assisted extraction and separation, alkaloid, pilot-scale, essential oil

## Abstract

Microwave extraction and separation has been used to increase the concentration of the extract compared to the conventional method with the same solid/liquid ratio, reducing extraction time and separate at the same time Volatile Organic Compounds (VOC) from non-Volatile Organic Compounds (NVOC) of boldo leaves. As preliminary study, a response surface method has been used to optimize the extraction of soluble material and the separation of VOC from the plant in laboratory scale. The results from the statistical analysis revealed that the optimized conditions were: microwave power 200 W, extraction time 56 min and solid liquid ratio of 7.5% of plants in water. Lab scale optimized microwave method is compared to conventional distillation, and requires a power/mass ratio of 0.4 W/g of water engaged. This power/mass ratio is kept in order to upscale from lab to pilot plant.

## 1. Introduction

Boldo (*Peumus boldus* Molina) is a dioic plant endemic in Chile [1]. Several pharmacopeias document its uses as leaves infusions for notably digestives and hepato-biliairy protective effects. These effects are caused by active molecules that can be classified as either Volatile Organic Compounds (VOC) or non-Volatile Organic Compounds (NVOC).

Among the active NVOC there are flavonoids, polyphenols [2–4] *i.e.*, quercetin, isorhamnetin, kaempferol, and alkaloids such as boldine which is the main alkaloid, a strong antioxidant and a free radical scavenger [5,6]. It is the main compound responsible for the digestive and liver protection effects. The boldine can be extracted by maceration in water. Boldo leaves contain also 0.4%–3% of essential oil, mainly composed of VOC, such as terpenes (ascaridole, *p*-cymene, *d*-limonene…). These molecules can have a low solubility in water, giving aqueous extract a distinctive odor. The presence of these VOC in the plant can raise several health concerns: they are toxic, irritant, or allergenic components [7–9]. The essential oil is bioactive, pro-oxidant and presents antifungal properties [4,10–12]. These VOC can easily be extracted from the leaves by azeotropic distillation.

Hydrodistillation is a very complete process. In one step, the VOC and the NVOC can be extracted and physically separated. The VOC are stripped from the matrix by azeotropic distillation, then condensed, collected, and separated in a Florentine flask. The soluble NVOC are extracted in the boiling water in contact with the matrix inside the alambic. However, hydrodistillation is highly energy- and time-consuming. Microwave assisted extraction (MAE) is a reliable alternative method of extraction. Compared to hydrodistillation, it consumes less energy and requires less time to carry out the extraction of essential oil [13,14].

We applied this microwave method in order to extract the different compounds of the boldo leaves in a single step, and to accelerate the hydrodistillation. The microwaves only heat the leaves and the water. As the matrix is also directly heated from the inside to the outside, it improves the extraction and solubilization of the leaves NVOC. The generated steam is also condensed to extract the essential oil from the leaves by using a Clevenger apparatus.

In this paper we report a study from lab to pilot scale for extraction and separation of volatile and non-volatile compounds from boldo leaves. We optimized experimental conditions (microwave power and time of extraction) by experimental plan design. The extraction yield of NVOC is monitored as well as the yield of VOC extracted in the process. Then, the optimization is used to demonstrate that this lab scale method can be performed in a pilote scale. The high performance thin layer chromatography (HPTLC) analysis of boldine served as a marker of interesting NVOC extracted; gas chromatography coupled to mass spectrometry (GC-MS) was used to identify and to have the relative proportion of each molecules in VOC extracted; to help us monitor the efficiency of the up scaling.

## 2. Results and Discussion

### 2.1. Characterization of the Conventional Method of Extraction

To evaluate the content of essential oil from the boldo leaves, a conventional hydrodistillation of 500 g of boldo leaves and 5 L of water was carried out. The VOC extraction stopped after 120 min of hydrodistillation. On an average of three repeated experiments, 11 mL of VOC are collected with a weight of 7.4 g. Our batch of boldo leaves had an average VOC content of 1.4%.

The NVOC were extracted with a yield of 24.90%. The extract displayed a concentration of NVOC and other soluble material of 2.83%.

The analysis of the boldo leaves extract showed a boldine content in leaves of 105.4 ppm.

### 2.2. Determination of Solid/Liquid Ratio

To determine a satisfactory ratio of boldo leaves and water, a series of experiments with the microwave apparatus were carried out to determine the solid/liquid ratio (*S*/*L*). With 500 mL of water in the vessel, we added from 5 to 50 g of boldo leaves. Microwave treatment is set at 900 W for 1 h.

**Figure 1.**
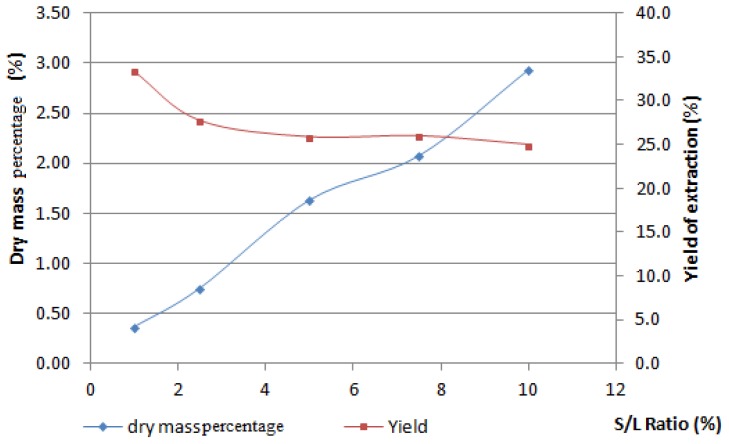
Soluble material extracted in function of Solid/Liquid ratio.

As seen from our previous study, 10% of solid/liquid ratio impairs the free movement of boldo leaves in the water during the experiment [15]. As we can see in Figure 1, the ratio of 7.5% offers a good dry mass ratio and yield of soluble material extraction. 7.5% of boldo leaves in the water is therefore chosen to undertake the experimental design studies. A higher ratio impairs the homogenization, and the swelling of the matrix traps non-negligible amounts of water.

### 2.3. Studies Using an Experimental Design

#### 2.3.1. Results for NVOC and VOC Yields

The coded and decoded values of independent variables and the responses obtained in the multivariate study for each experiment are shown in Table 1. Time and microwave power are evaluated, with NVOC and VOC Yields as responses.

**Figure 2.**
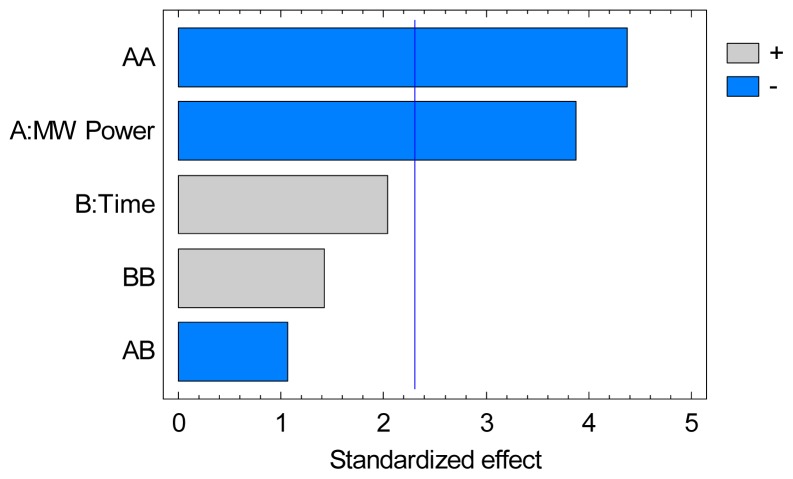
Standardized Pareto chart of optimization multivariate study of NVOC Yield.

In this second part of the study, the effect microwave power and time of extraction on the yields of soluble material in the extract and on the VOC yield separated from the product were evaluated by response surface methodology. ANOVA for NVOC yield gave a coefficient of determination (*R*^2^) of 83.2%, which indicates a close agreement between experimental and predictive values. It can be explained by the very close results between all the experiments. ANOVA data summarized in a Pareto chart in Figure 2 for NVOC yield represents the significant effects of all variables (linear and quadratic) and their interactions. It can be seen that the quadratic effect of microwave power, as well as microwave power, have the most significant influence on the NVOC yield. This suggests a maximal microwave power value useable for optimal extraction. It is followed with less significant influence by time. The lack of significance of the cross-product terms suggests the absence of interactions between variables in the studied range. The experimental data obtained from the CCF allowed us to determine an empirical relationship linking response studied (Yield) and key variables involved in the model (in coded units). Thus, a 2nd order polynomial equation was obtained Equation (1). The large constant value suggests that most NVOC extraction occurs within the first 20 min of microwave treatment.

(1) $$NVOC\;Yield=23.09+0.0092\times MWPower-0.0000089\times MWPower^{2}$$

**Figure 3.**
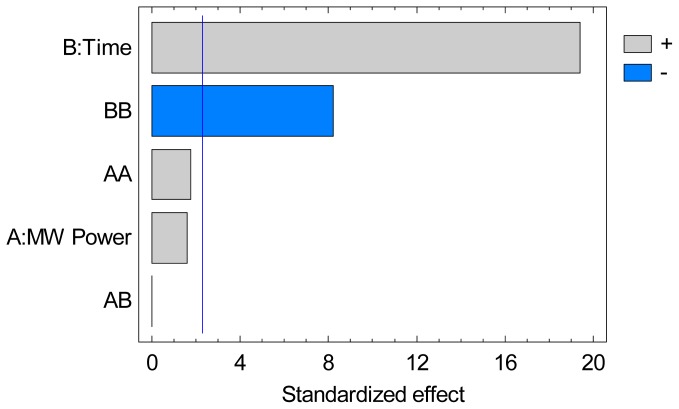
Standardized Pareto chart of multivariate response for VOC yield.

ANOVA data are summarized in a Pareto chart in Figure 3 for the VOC yield, gave 98.2% as coefficient of determination (*R*^2^) which indicates a good agreement between experimental and predictive values. The effect of time is majorly influencing the results followed by its quadratic effect, showing that a plateau was reached for the extraction of VOC. Both the linear and quadratic effects of microwaves power lie below the level of significant influence. This means that only time is significant in the separation process of the VOC and the quadratic effect of time suggest a final time of extraction exists. The experimental data obtained from the CCF allowed us to determine an empirical relationship linking response studied (VOC Yield) and key variables involved in the model (in coded units). Thus, a 2nd order polynomial equation was obtained Equation (2):

(2) $$VOC\;Yield=-0.50+0.069\times Time-0.00062\times Time^{2}$$

#### 2.3.2. Optimization of Yield of Extraction and Separation of VOC and NVOC

**Figure 4.**
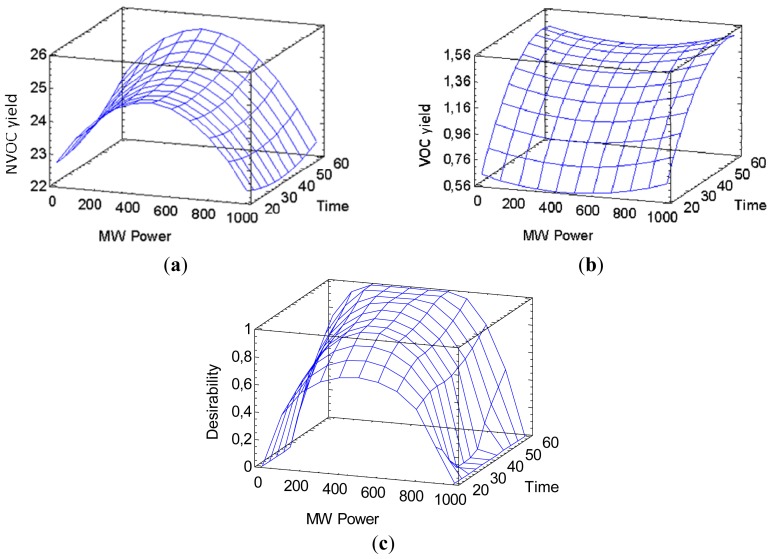
Optimization of microwave assisted extraction of boldo leaves in water: (**a**) NVOC yield (%) investigation in the multivariate study as a function of microwave power and time; (**b**) VOC yield (%) as investigation in the multivariate study as a function; (**c**) Response surface of optimization between yield and essential oil separation.

The parameters optimization was done thanks to the combined data of NVOC and VOC yields. We want to maximize NVOC yield as well as the VOC separation. NVOC yield can be increased slightly by increasing time, but can be degraded by higher microwave power as seen in Figure 4a). VOC separation is increased as time increase and hit a maximum. It is however not influenced by power in a significant level as we can see in Figure 4b).

As we aim for a green extraction, the less energy consumed the better; therefore, an optimum with both low time and power for high NVOC and VOC yields must be reached. We obtained ebullition in only 15 min.

By combining the two results, we can calculate an optimal set of conditions for the best extraction process of soluble material and the complete separation of VOC. The software calculates this optimum set of conditions at 200 W and 56 min of extraction, with a predicted NVOC yield of 25% and 1.36% of VOC. These predictions can be modeled as a surface in Figure 4c).

### 2.4. From Lab Scale to Pilot Scale

For the purpose of this study, the scale up factor is calculated with a constant ratio between microwave power and mass of water involved. The optimum conditions required 200 W for 500 mL of water, the maximum power output of the MAC-75 of 6 kW need 15 L of water to work. A total weight of 1.125 kg of boldo leaves is processed. This represents a scale-up factor of 30. The microwave system was set at maximum power for 56 min and ebullition was obtained in 15 min. As seen in Table 2, the amount of essential oil extracted from the boldo leaves is equivalent to the lab scale method. Similarly, the extract quality is identical to lab scale extraction in terms of yield and also of boldine content.

Furthermore, the quality and quantity extracted from boldo by microwave are greater than conventional hydrodistillation, with nearly a third of the time and a quarter of the energy in order to extract it.

The reduced cost of extraction provided by our proposed MAE method relies on the reduced consumption of energy and time. As hydrodistillation required 30 min to start the VOC azeotropic distillation, where the MAE only needed 5 min. This proved the efficiency of heating energy delivered in the matrix.

### 2.5. Analysis of the Essential Oil

A total of 40 VOC were identified in boldo essential oils using the three extractions techniques. Their nature and relative amounts are reported in Table 3. MAE lab and pilot scale as well as hydrodistillation enabled the detection of most volatile active compounds in boldo essential oil such as *p*-cymene, *d*-limonene, and ascaridole. However, their proportions vary with the extraction technique. Lower amount of oxygenated compounds are present in the essential oils of the boldo extracted by the two MAE compared with HD. *p*-cymene is better extracted in MAE lab and scale up than HD. The *d*-limonene is present in equivalent amounts in lab scale MAE and HD, with a higher amount in scale up MAE. The essential oil from boldo leaves isolated by all techniques contains the same dominant components. Ascaridole, an oxygenated monoterpene, is the main abundant component in the essential oil with increasing relative amounts for the three extractions methods, 36.51%, 40.94%, and 46.90%, respectively, for scale up MAE, lab scale MAE and HD. This suggests that a small part of this compound remains inside the leaves.

The difference in relative proportion between the different compounds could be explained by the time difference of essential oil extraction, where in MAE it is not long enough to allow the most difficult molecules to be completely extracted such as the oxygenated molecules [16].

### 2.6. Sensorial Analysis

**Figure 5.**
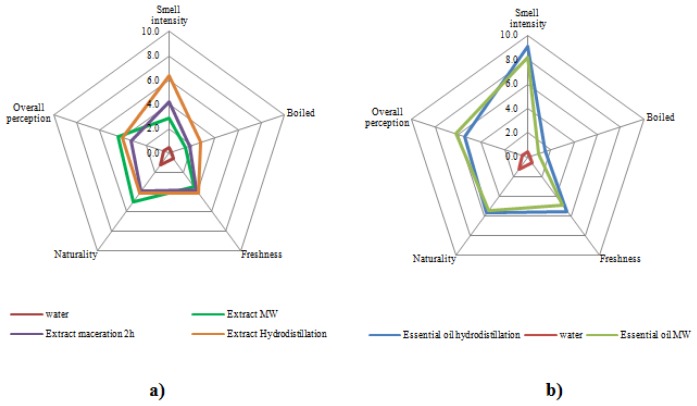
Sensorial analysis of: (**a**) aqueous extracts and (**b**) essential oils.

The results of sensorial tests show that the extracts from hydrodistillation and microwave extraction have far lower smell intensity than maceration, with a lighter smell with microwave as seen in Figure 5a. The microwave extract quality is also improved in comparison to hydrodistillation and maceration as it is less perceived as “boiled”, has a better natural quality, keeps its freshness and is generally preferred to the others. In Figure 5b, we can observe that the two different essential oils are very closely related, with a slightly better fragrance and a lesser boiled smell for microwave than with hydrodistillation. It is also noted a slight smell difference between the two of them.

## 3. Experimental Section

### 3.1. Plant Material and Chemicals

Boldo leaves were collected in May 2012, they contain 5% moisture. Chloroform HiPerSolv chromanorm, diethylamine GPR rectapur, chlorhydric acid 35% rectapur come from VWR Prolabo (Darmstadt, Germany). The toluene analytical reagent is provided by Fisher (Illkirch, France). The methanol HPLC for Gradient Analysis is provided by Acros Organics (Slangerup, Denmark). Ammonia puriss is bought to Sigma Aldrich (St. Louis, MO, USA). The standard boldine for HPTLC and degradation study comes from Extrasynthèse (Genay, France).

### 3.2. Extraction Procedures

**Figure 6.**
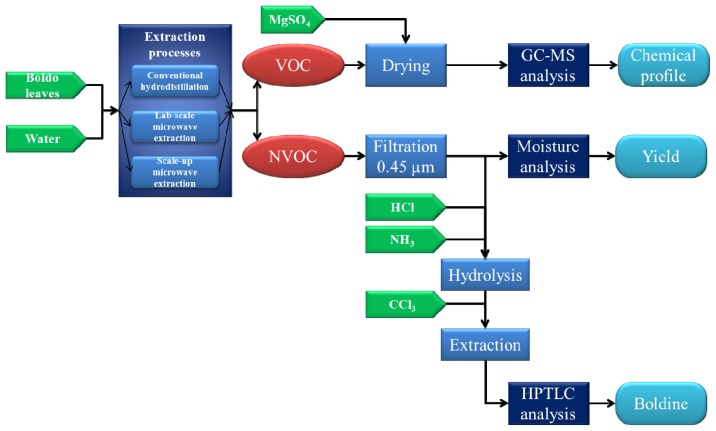
Protocol treatments of boldo leaves.

The boldo leaves undergone two operations simultaneously: the extraction of VOC and NOVC, and theirs separations. Both extracts are aliquoted and analyzed using different methods. All processes of extraction, separation and analysis can be followed in Figure 6.

#### 3.2.1. Microwave Assisted Extraction (MAE)

**Figure 7.**
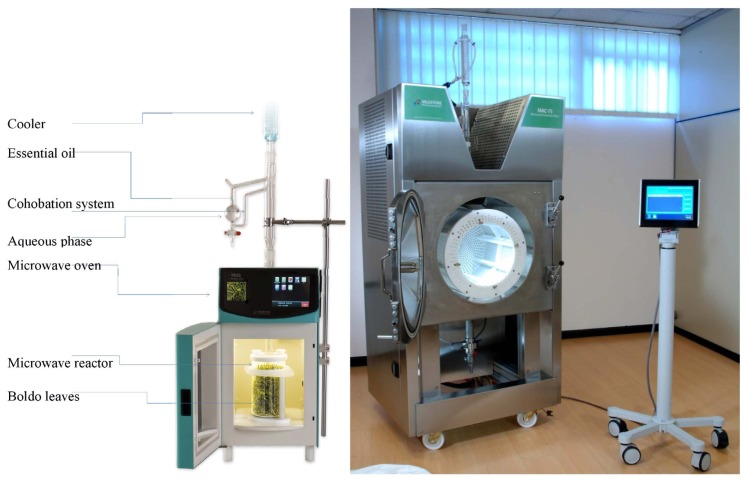
Laboratory and pilot scale microwave extractor.

Microwave Assisted Extraction (MAE) was performed in Milestone EOS-G microwave laboratory oven illustrated in Figure 7. This apparatus is a multimode microwave reactor 2.45 GHz with a maximum delivered power of 900 W variable in 10 W increments. The 1 L extraction vessel is made of Pyrex. A Clevenger system is used to collect the condensed vapors, collect the essential oil and return the distillated water in the extraction vessel.

MAE procedure was performed at atmospheric pressure. A fixed volume of 500 mL of distillated water was used with the selected amount of boldo leaves, determined by solid/liquid ratio optimization. They are added in the extraction vessel. The time and power of microwaves are selected. Once the extraction is done, VOC are collected from the Clevenger, dried on magnesium sulfate, filtered and weighted on an analytical balance within a 1% error margin. VOC collected are stored in amber glass vial at 4 °C until used. The extract and leaves mixture are filtered on a sieve. Boldo leaves are pressed to gather all extract. An aliquot of the extract is made for analysis, filtered on 0.45 μm PTFE filter stored in amber glass vial at −20 °C until used.

#### 3.2.2. Conventional Hydrodistillation (HD)

The conventional hydrodistillation was performed in a stainless-steel alembic set with a Clevenger system [17] according to the European Pharmacopoeia. 500 g of boldo leaves were submitted to hydrodistillation with 5 L of distillated water for 146 min (until no more essential oil was obtained). This apparatus is then used with the optimal conditions found after the experimental design on the Microwave Assisted Extraction (MAE) for comparison.

Volatile Organic Compounds (VOC) are collected, dried on magnesium sulfate, weighted on an analytical balance within 1% error margin and stored in amber glass vial at 4 °C until used.

#### 3.2.3. Scale up Microwave Assisted Extraction

The MAC-75 apparatus as seen in Figure 7 is a multimode microwave reactor. It contains four magnetrons (4 × 1500 W, 2450 MHz) with a maximum power of 6000 W delivered in 500 W increments. The stainless steel microwave cavity has a capacity of 150 L and contains a removable, rotating PTFE drum that allows up to 75 L of plant material to be loaded. The rotation ensures a homogeneous microwave distribution to the material inside the drum. The drum circumference is entirely perforated to allow the vapor and liquid to pass. The absorption of microwave power is controlled by sensors placed on wave guides. The system automatically adjusts the power delivered if absorption is too low.

The temperature is monitored by a Resistance Temperature Detector (RTD, PT-100) inserted into the cavity. The main difference is that the RTD changes its electrical resistance in response of temperature changes, the thermocouple produce very low voltage changes between two metallic materials in response of temperature changes. Both sensors can be used in a microwaves system using some cautions, but using RTD is easier due to its construction. The measurement problems that could happen using a temperature sensor in microwave field are the following:

- The microwaves reach the sensing element of the sensor, producing an electrical noise resulting in a mismeasurement.
- The microwaves electrically charge the metallic container of the sensor up to very high voltage, producing sparks near to the sensors tip. The temperature can increase locally due to the sparks.

The use of RTD in our microwave device is designed to avoid both side-effects described above:

- The RTD we use is fully embedded in a metallic container (Stainless steel AISI 316) and is a perfect Faraday’s cage. In this way the microwaves do not reach the sensing element at all.
- The metallic container of the sensor is grounded, the tip is shorter than microwaves wavelength and recessed in a metallic shield of specific length and diameter. This construction is a microwaves filter: the 2450 MHz generated cannot electrically charge the metallic container of the sensor to high voltage. In this way the microwaves do not produce sparks on the sensor surface.

The RTD we use has a response time lower than 5 s (in accordance with IEC 751), with ±0.3 °C of error. This error must be added to the electronic analog to digital converter error that is less than 1 °C.

The cavity is able to work in deep vacuum or as an open vessel. Interlocks on the door prevent accidental opening during the process or when the cavity contains liquid. The device is controlled by an industrial touch screen control terminal with an intuitive graphic user interface.

The plant material mass determined by optimization and calculated by the appropriate scale-up factor is weighted and loaded in a neutral fiber bag. The set amount of water is loaded in the microwave cavity. The top opening is set up with a condenser to collect the vapors and direct them in a Florentine flask to separate distillate and essential oil. The microwave apparatus is programmed with the time and power selected. After distillation, VOC are collected, dried on magnesium sulfate and weighted on an analytical balance within 1% error margin, stored in amber glass vial at 4 °C until used. The extract in the microwave cavity is collected, and an aliquot is made for analysis, filtered on 0.45 μm PTFE filter, stored in amber glass vial at −20 °C until used.

### 3.3. Analysis of Extracts

#### 3.3.1. Dry Mass Percentage

Dry Mass percentage (%DM) is determined by using a moisture analyzer (OHAUS MB35, OHAUS, Nänikon, Switzerland). 5 g of 0.45 μm PTFE filtered sample are heated 45 min at 110 °C to obtain a stable mass. This method gave us the water content of the extract, therefore also the NVOC content of the extract Equation (3). Therefore, the yield of NVOC is calculated as follows: Equation (4).

(3) %DM=100-moisture percent of extract

(4) $$NVOC\;yield=\%DM\times \frac{mass\;of\;extract}{dry\;mass\;of\;boldo\;leaves}$$

The measurements are within a 2% error margin.

#### 3.3.2. Isolated Compound Study

In order to investigate whether the boldine present in the extract is likely to undergo degradation during the microwave irradiation, a solution of boldine is submitted to microwave treatment. With a final concentration of 0.02 mg/mL, the solution is placed in the glass reactor, followed by a microwave treatment under the conditions determined as optimal in the design of experiments. One sample untreated and one sample treated by microwaves was then analyzed by HPTLC for quantification. All analyses are carried out in triplicate.

#### 3.3.3. Boldine Analysis

Five grams of 0.45 μm filtered extract were acidified with 0.5 mL of hydrochloric acid (6 M, Rectapur, VWR Prolabo; Darmstadt, Germany) at 100 °C for 10 min. Then, after cooling at 20 °C, the mixture was basified with an ammonium solution of 1 mL at 25% (Puriss, Sigma Aldrich; St Louis MO, USA). The hydrolyzed sample was extracted with 5 mL of chloroform (HiPerSolv Chromanorm, VWR Prolabo; Darmstadt, Germany) and both phases are placed in a tube for centrifugation (at 1500 × *g* for 5 min). The organic phase was collected and dried at 45 °C under a nitrogen stream. The dry extract was solubilized with 5 mL of chloroform, filtered on 0.45 μm PTFE syringe filter (VWR Prolabo; Darmstadt, Germany) put in vial for High Performance Thin Layer Chromatographic run (HPTLC).

The samples and standard were spotted in the form of bands of width 8 mm with a Camag microliter syringe controlled by the Automatic TLC Sampler ATS 4 (Camag; Muttenz, Switzerland) on precoated silica gel glass Plate 60 Å F254 (20 × 10 cm; Merck, Darmstadt, Germany). The plates were prewashed by propan-2-ol and activated at 120 °C for 20 min prior to spotting. A constant application rate of 200 nL/s was employed and space between two bands was 8.5 mm.

The migration of the plate was carried out in 20 × 10 cm twin through glass chamber of an Automatic Development Chamber ADC2 (Camag; Muttenz, Switzerland). The plate is first dried for 5 min to evaporate any residual solvent from the samples and standards. The mobile phase consisted of toluene-methanol-diethylamine (40:5:5, *v*/*v*/*v*) and 10 mL of mobile phase were used per chromatography. The glass chamber was saturated for 10 min at 25 °C and at relative humidity of 45% with an additional 10 mL of mobile phase. The Thin Layer Chromatography (TLC) plate is pre-saturated for 10 min. The length of chromatography run is of 60 mm to allow results in better apparent resolution conveniently within the capability of the detecting device to perform integration of peak area. Subsequent to the development, TLC plates were dried in a current of air from the ADC2.

The densitometric analysis was performed on CAMAG TLC Scanner 3 (Camag; Muttenz, Switzerland). The slit dimension was kept 6.00 × 0.10 mm and 10 mm/s Scanning speed was employed. The scanning was performed at 307 nm in reflectance/absorbance mode. The source of radiation used was Deuterium/Tungsten lamp emitting a continuous UV spectrum between 190 and 400 nm. Each track is scanned and baseline correction is used. All operations were monitored by WinCATS software (V 1.4.7.2018, Camag; Muttenz, Switzerland). Standard of boldine were prepared by solubilizing 10 mg of boldine (Extrasynthèse, Genay, France) in 500 mL of chloroform. Five spots of the standard of increasing volume were made to obtain concentration rage of 50–300 ng/spot. The median concentration of the range was repeated five times on the TLC plate.

### 3.4. Gas Chromatography and Mass Spectrometry Analysis

VOC composition was determined by gas chromatography coupled to mass spectrometry (GC-MS) analysis on a Agilent 7890A gas chromatograph coupled to a 5975C Mass spectrometer, using a fused-silica-capillary non polar column: a HP5MS™ (30 m × 0.25 mm × 0.25 μm film thickness; Agilent, Kyoto Japan). GC-MS spectra were obtained using the following conditions: carrier gas He; flow rate 0.7 mL/min; split 1:200; injection volume 0.5 μL; oven temperature progress from 3 min at 60 °C then to 90 °C at 3 °C/min, then to 115 °C at 1 °C/min, hold for 1 min, then to 240 °C at 5 °C/min; the ionization mode used was electronic impact at 70 eV. Most constituents were tentatively identified by comparison of their retention indices (RI), determined with reference to an homologous series of C_5_–C_24_ *n*-alkanes and with those of authentic standards available in the authors’ laboratory. Identification was confirmed by comparison of their mass spectral fragmentation patterns from the software AMDIS (Automatical Mass spectral Deconvolution and Identification System by the National Institute of Standards and Technology USA), internal and public mass spectrum libraries and scientific literature. The quantitative analysis of the components is given is percent of the total chromatographic peaks area by setting the hypothesis: the response factor is identical for all molecules.

### 3.5. Experimental Design

Box-Wilson design, also called Central Composite Design (CCD), was used to achieve optimal conditions of the process with a minimal number of possible experiments. The type of CCD used in this study was central composite face-centered (CCF) experimental design to determine the optimal conditions of MAE. The application of a CCF design is a convenient way to optimize a process with five levels (−α, −1, 0, +1, and +α) for each factor. In this design, the star points are at the center of each face of the factorial space, thus ±α = ±1. This design is needed to evaluate the effects and interactions effects of two independent variables, namely time of extraction (*t*), and power (*P*). The microwave irradiation power was varied between 200 and 900 W whereas the time of extraction varies from 20 to 60 min. A total of 12 different combinations, including 2^2^ full factorial design (±1) with four axial points (±α) and four replicates of center point (coded 0), chosen in random order according to a CCF configuration for two factors, was employed for response surface modeling.

The selected optimization responses were the yield of Non Volatile Organic Componds and Volatile Organic Compounds yields. The experimental designs used were constructed and the experimental results were processed by using the software STATGRAPHICS PLUS (Version 15.01.02, Statistical Graphics Corporation, Rockville, MD, USA, 2000). An analysis of variance (ANOVA) with 95% confidence level was then carried out for each response variable in order to test the model significance and suitability. The *F*-value in ANOVA is the ratio of mean square error to the pure error obtained from the replicates at design center and the *p*-value defines the significance of the different variables. A description of significant effects obtained from ANOVA for extraction time *t* (min) was presented by a Standardized Pareto Chart.

### 3.6. Sensorial Analysis

The sensory analysis of five samples was conducted by a panel consisting of 15 members from Avignon University, Avigon, France (water, extracts from conventional hydrodistillation, lab scale MAE and maceration; essential oils from hydrodistillation and microwave extraction). The subjects were seated in sensory booths with appropriate ventilation and lighting. The anonymous samples were presented to each panelist in amber glass bottles. For the five samples, the following attributes were evaluated: strength, boiled, freshness, naturalness, and global acceptance. For overall quality, the scale ranged from 0 (weakest attribute) to 10 (strongest attribute) and a score of 5 corresponded to an ideal perception. The panelists gave their preferences for each sample on a hedonic scale (0–10). The average of the points was calculated for each attribute.

## 4. Conclusions

The proposed Microwave Assisted Extraction system is more efficient in the aqueous extraction of boldo leaves and the essential oil separation than the conventional hydrodistillation. The experimental design allowed us to optimize the parameters of the extraction in order to maximize the extraction yield and essential oil separation while decreasing time and power consumption. The final extract is shown to have similar composition and olfactive properties as its lab scale equivalent. The use of microwave also improves the sensorial properties of the extract while decreasing the strength of its smell, allowing a better integration in possibly perfumed cosmetic formulation.

The scale up to pilot scale is shown as possible on a factor 30 by the use of MAC-75 microwave apparatus and was confirmed by another study with the solvent free extraction of essential oil from rosemary [18], where it was extracted with a better efficiency in the MAC-75 with less time and less energy than conventional hydrodistillation. Even though the scale up process parameters was not optimized for the apparatus in our study, these results are promising. Further transpositions of the lab scale to pilot scale using the MAC-75 are underway along with the optimization of this new equipment.

The process is more sustainable, with a greater efficiency in energy use and less time-consuming. This is confirmed by other studies of microwave assisted extractions made in the past [19–22]. No additional water was needed in order to extract and separate VOC during the extraction of NVOC. It also displays an improved safety, is allowing simplified handling operations to load and unload the vegetal matrix and solvent. Thanks to an additional removable drum and multiple fiber bags, the process can be easily quickly stopped, cleaned and reloaded for a new run. This extraction method combined with this new microwave apparatus indicates potential for industrial use for day-to-day operations but also fit in a process to create an equipment of continuous extraction.

## Figures and Tables

**Table 1.** Variables involved in the Central Composite Design (CCD) and response obtained.

Time (min/coded)		Power (W/coded)		NVOC Yield (%)	VOC Yield (%)
20	−1	200	−1	24	0.6
40	0	200	−1	24.6	1.2
60	1	200	−1	25	1.4
20	−1	550	0	24.9	0.6
40	0	550	0	24.1	1.2
60	1	550	0	25.9	1.4
20	−1	900	1	23.1	0.6
40	0	900	1	23.3	1.4
60	1	900	1	23.2	1.4
40	0	550	0	25.1	1.2
40	0	550	0	24.7	1.2
40	0	550	0	24.2	1.2
40	0	550	0	24.7	1.2
40	0	550	0	24.9	1.2

**Table 2.** Comparison between the different extractions procedures.

Results	Lab Scale MAE	Conventional Hydrodistillation	Scale up MAE
Dry mass percentage (%)	2.11	2.83	2.10
NVOC yield (%)	25.67	24.90	26.1
VOC yield (%)	1.4	1.4	1.4
Boldine (ppm)	122.4	105.4	129
Total time of extraction (min)	56	146	56
Energy consumption (J/g boldo)	1344	5256	1344

**Table 3.** Chemical composition of boldo essential oils obtained by hydrodistillation (HD), microwave assisted extraction (MAE), and scale up MAE.

N°	Compounds a	HD (%)	MAE Scale up (%)	Lab Scale MAE	RI b
	**Monoterpene hydrocarbons**	**35.8**	**47.1**	**40.4**	
1	Thujene alpha	0.1	0.1	0.2	924
2	Pinene alpha	1.0	0.9	1.2	931
3	Camphene	0.2	0.2	0.2	945
4	Sabinene	1.0	1.3	1.1	971
5	Pinene beta	0.4	0.5	0.5	974
6	Myrcene beta	0.2	-	0.2	990
7	Phellandrene alpha	-	-	0.1	1004
8	3-Carene	0.2	0.2	0.2	1009
9	Alpha. Terpinene	0.3	0.4	0.4	1015
10	Para. Cymene	12.9	19.0	16.8	1026
11	*d*-Limonene	18.8	23.2	18.5	1033
12	Gamma. Terpinene	0.4	0.7	0.6	1057
13	Terpinolene	0.4	0.5	0.4	1087
	**Oxygenated monoterpenes**	**57.3**	**47.4**	**50.4**	
14	Linalol	1.0	1.6	0.9	1101
15	Fenchol	0.2	0.3	0.2	1111
16	Cis, para. 2-Menthen-1-ol	0.4	0.3	0.3	1118
17	Campholenic Aldehyde	-	0.1	-	1122
18	Para. 2,8-Menthadien-1-ol, *cis*	0.1	-	0.1	1131
19	Trans Pinocarveol	0.6	0.6	0.5	1133
20	Trans-p-2-menthen-1-ol	0.1	0.2	0.1	1135
21	Camphor	0.2	0.2	0.1	1138
22	Sabina ketone	0.2	0.2	0.2	1152
23	Pinocarvone	0.6	0.9	0.7	1156
24	Borneol	-	0.1	-	1160
25	1-Terpinen-4-ol	2.2	2.0	1.7	1173
26	Cryptone	0.3	0.2	0.3	1181
27	Alpha-terpineol	2.2	1.6	1.4	1188
28	Myrtenal	0.5	0.7	0.6	1192
29	Ascaridole	46.9	36.5	40.9	1242
30	Dérivé Ascaridole	0.3	0.5	-	1252
31	Thymol	0.3	0.3	0.4	1288
32	Carvacrol	0.2	0.2	0.3	1297
33	Methyl Eugenol	0.9	0.8	1.1	1405
34	Spathulenol	0.4	0.3	0.7	1572
	**Oxygenated sesquiterpenes**	**0.2**	**0.3**	**1.0**	
35	Nerolidol (E)	-	-	0.2	1568
36	Caryophyllene oxide Isomer 1	-	0.1	0.2	1575
37	Viridiflorol	-	-	0.2	1592
38	Alpha.-Cadinol	-	-	0.1	1649
39	Alpha.-Bisabolol	0.2	0.2	0.4	1681
	**Other oxygenated compound**	**0.2**	**0.3**	**0.3**	
40	Bornyl acetate	0.2	0.3	0.3	1282
	**Extraction time (min)**	**150.0**	**56.0**	**56.0**	
	**Yield (%)**	**1.4**	**1.4**	**1.4**	
	**Total oxygenated compounds**	**57.8**	**48.0**	**51.7**	
	**Total non oxygenated compounds**	**35.8**	**47.1**	**40.4**	

a Essential oil compounds sorted by chemical families;

b Retention indices relative to C5–C24 n-alkanes calculated on non-polar HP5MS capillary column.
